# Supplementary material for: Inducible MLL-AF9 Expression Drives an AML Program during Human Pluripotent Stem Cell-Derived Hematopoietic Differentiation
Source: Cells. 2023 Apr 20;12(8):1195. doi: 10.3390/cells12081195 (PMC10136707; doi:10.3390/cells12081195)
Supplement: Supplementary file 1 [file cells-12-01195-s001.zip › 230419_Supplementary_Figures.pdf]

Inducible MLL-AF9 expression drives an AML program during human pluripotent stem cell-derived hematopoietic differentiation

Branco M. H. Heuts[1,+], Saioa Arza-Apalategi[2,+], Sinne G. Alkema[1], Esther Tijchon[1], Laura Jussen[1], Saskia M. Bergevoet[2], Bert A. van der Reijden[2,+,*], Joost H. A. Martens[1,+,*]

1 Radboud University, Faculty of Science, Department of Molecular Biology, 6525 GA Nijmegen, The Netherlands.

2 Radboud University Medical Center, Department of Laboratory Medicine, Laboratory of Hematology, 6525 GA Nijmegen, The Netherlands.

* bert.vanderreijden@radboudumc.nl, joost.martens@ru.nl

+ these authors contributed equally to this work.

Figure S1

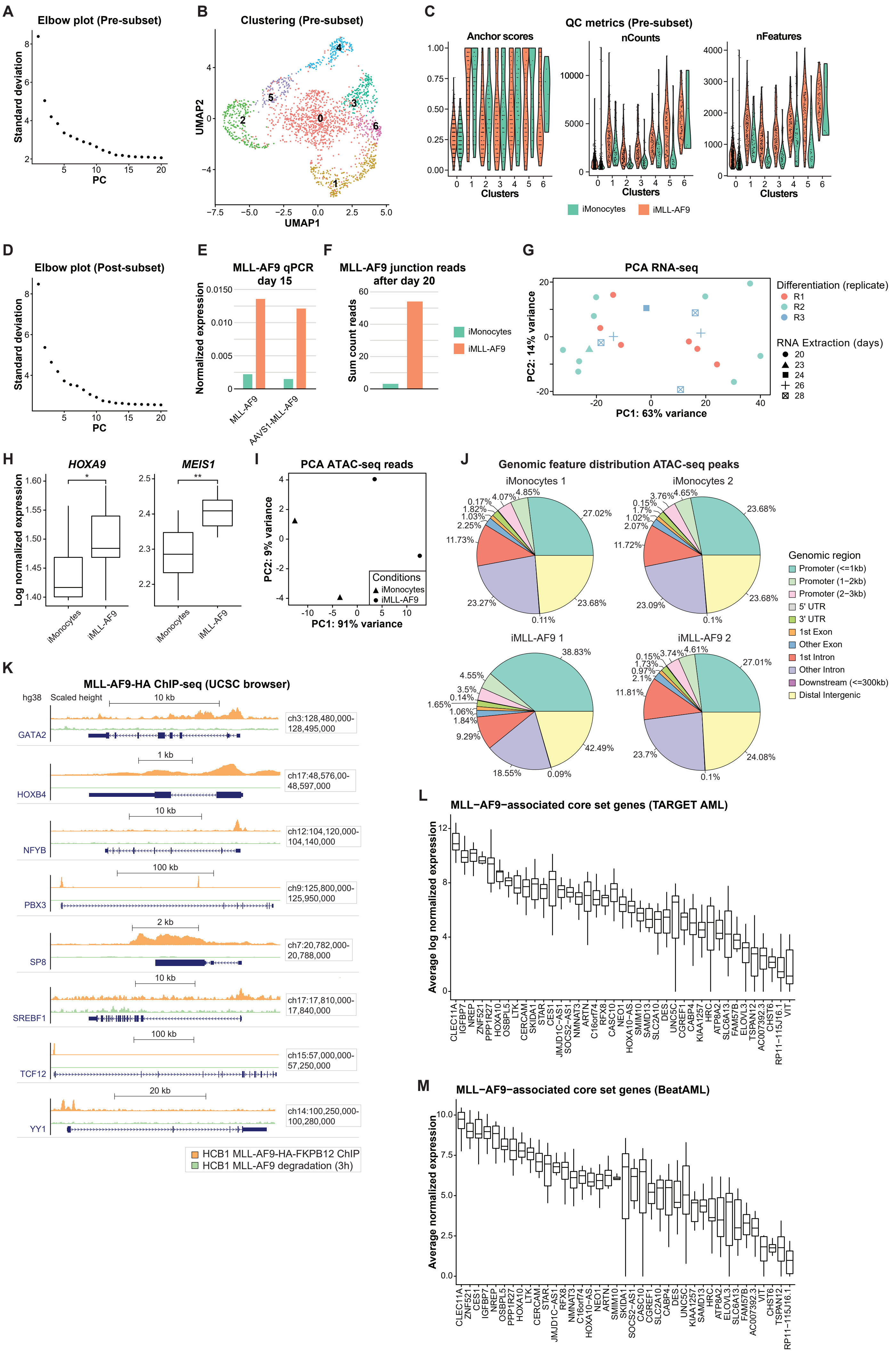
A
Elbow plot (Pre-subset)
Standard deviation
PC
B
Clustering (Pre-subset)
UMAP1
UMAP2
C
QC metrics (Pre-subset)
Anchor scores
nCounts
nFeatures
Clusters
iMonocytes
iMLL-AF9
D
Elbow plot (Post-subset)
Standard deviation
PC
E
MLL-AF9 qPCR
day 15
Normalized expression
MLL-AF9
AAVS1-MLL-AF9
F
MLL-AF9 junction reads
after day 20
Sum count reads
iMonocytes
iMLL-AF9
G
PCA RNA-seq
PC1: 63% variance
PC2: 14% variance
Differentiation (replicate)
R1
R2
R3
RNA Extraction (days)
20
23
24
26
28
H
HOXA9
MEIS1
Log normalized expression
iMonocytes
iMLL-AF9
I
PCA ATAC-seq reads
PC1: 91% variance
PC2: 9% variance
Conditions
iMonocytes
iMLL-AF9
J
Genomic feature distribution ATAC-seq peaks
iMonocytes 1
iMonocytes 2
iMLL-AF9 1
iMLL-AF9 2
Genomic region
Promoter (<=1kb)
Promoter (1−2kb)
Promoter (2−3kb)
5' UTR
3' UTR
1st Exon
Other Exon
1st Intron
Other Intron
Downstream (<=300kb)
Distal Intergenic
K
MLL-AF9-HA ChIP-seq (UCSC browser)
hg38
Scaled height
GATA2
ch3:128,480,000-128,495,000
HOXB4
ch17:48,576,00-48,597,000
NFYB
ch12:104,120,000-104,140,000
PBX3
ch9:125,800,000-125,950,000
SP8
ch7:20,782,000-20,788,000
SREBF1
ch17:17,810,000-17,840,000
TCF12
ch15:57,000,000-57,250,000
YY1
ch14:100,250,000-100,280,000
HCB1 MLL-AF9-HA-FKPB12 ChIP
HCB1 MLL-AF9 degradation (3h)
L
MLL−AF9−associated core set genes (TARGET AML)
Average log normalized expression
M
MLL−AF9−associated core set genes (BeatAML)
Average log normalized expression

Figure S2

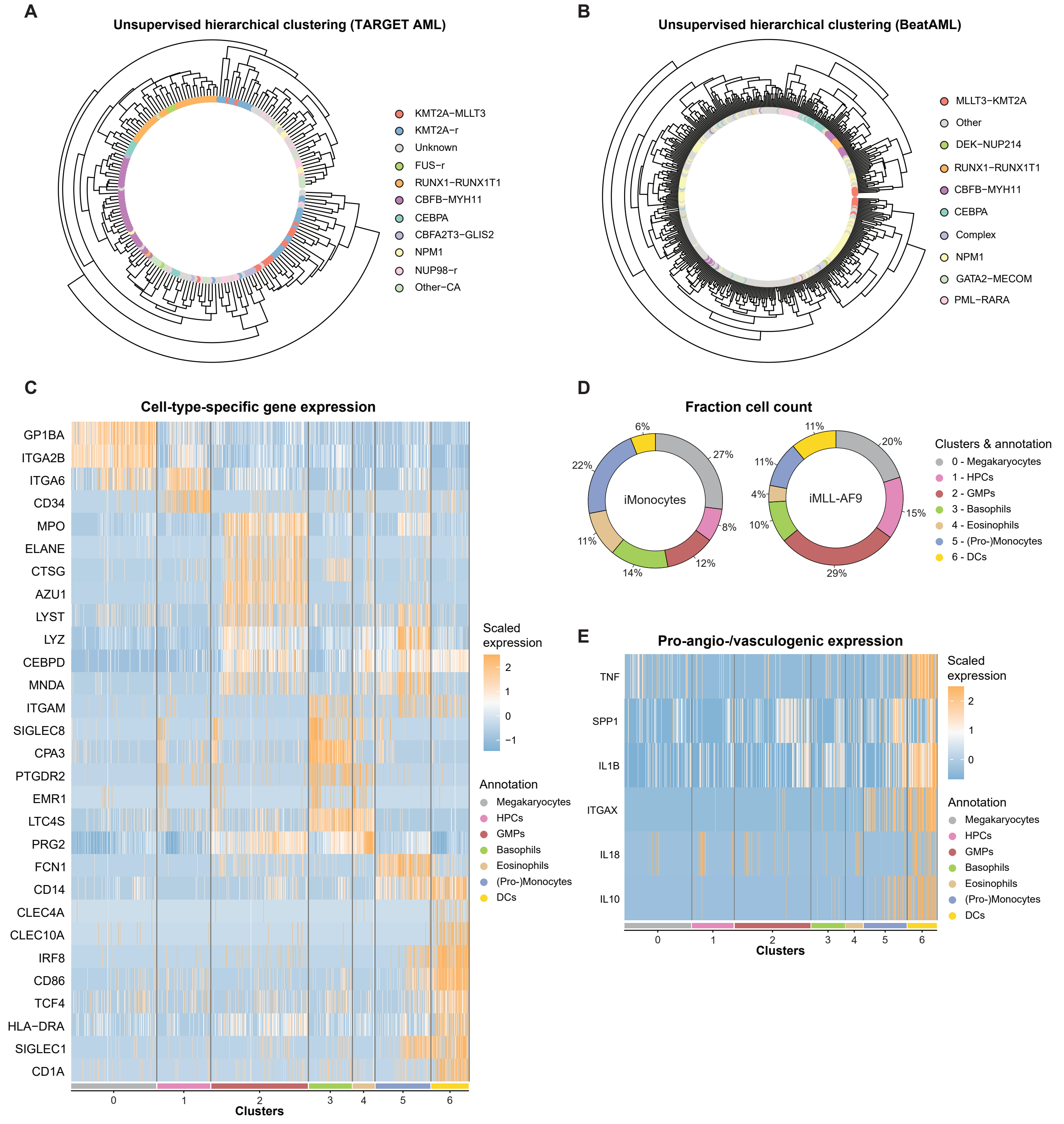
A
Unsupervised hierarchical clustering (TARGET AML)
KMT2A−MLLT3
KMT2A−r
Unknown
FUS−r
RUNX1−RUNX1T1
CBFB−MYH11
CEBPA
CBFA2T3−GLIS2
NPM1
NUP98−r
Other−CA
B
Unsupervised hierarchical clustering (BeatAML)
MLLT3−KMT2A
Other
DEK−NUP214
RUNX1−RUNX1T1
CBFB−MYH11
CEBPA
Complex
NPM1
GATA2−MECOM
PML−RARA
C
Cell-type-specific gene expression
GP1BA
ITGA2B
ITGA6
CD34
MPO
ELANE
CTSG
AZU1
LYST
LYZ
CEBPD
MNDA
ITGAM
SIGLEC8
CPA3
PTGDR2
EMR1
LTC4S
PRG2
FCN1
CD14
CLEC4A
CLEC10A
IRF8
CD86
TCF4
HLA−DRA
SIGLEC1
CD1A
Scaled expression
2
1
0
−1
Annotation
Megakaryocytes
HPCs
GMPs
Basophils
Eosinophils
(Pro-)Monocytes
DCs
0
1
2
3
4
5
6
Clusters
D
Fraction cell count
iMonocytes
6%
27%
22%
8%
11%
12%
14%
iMLL-AF9
11%
20%
11%
4%
15%
10%
29%
Clusters & annotation
0 - Megakaryocytes
1 - HPCs
2 - GMPs
3 - Basophils
4 - Eosinophils
5 - (Pro-)Monocytes
6 - DCs
E
Pro-angio-/vasculogenic expression
TNF
SPP1
IL1B
ITGAX
IL18
IL10
Scaled expression
2
1
0
Annotation
Megakaryocytes
HPCs
GMPs
Basophils
Eosinophils
(Pro-)Monocytes
DCs
0
1
2
3
4
5
6
Clusters

Supplementary figure legends

Supplementary Figure S1. Single-cell RNA-seq QC, MLL−AF9−associated gene expression, ATAC-seq QC, UCSC regions of predicted important TFs of iMLL-AF9, and unsupervised hierarchical clustering using only MLL−AF9−associated core set genes.

(**A**) Elbow plot visualizing the standard deviation of each PC (pre-subset dataset).

(**B**) UMAP visualizing Louvain clusters (pre-subset dataset).

(**C**) Violin plots depicting anchors scores, number of features, and number of counts for each cluster. The color represents the conditions.

(**D**) Elbow plot visualizing the standard deviation of each PC (post-subset dataset).

(**E**) Bar charts representing MLL-AF9 qPCR normalized to GAPDH. Two primer pairs were used for the qPCR (MLL-AF9 and AAVS1-MLL-AF9). Green color depicts iMonocytes and orange the iMLL-AF9 cells.

(**F**) Bar charts representing summed junction reads mapped to MLL-AF9 after day 20. Colors represent the doxycycline treatment conditions.

(**G**) PCA plot of iMonocytes and iMLL-AF9 cells. RNA-seq samples collected from three independent differentiation experiments, including at least three biological replicates each between day 20 and day 28. Colors represent the three different differentiation experiments that were used for analysis. Shapes depict the days at which RNA was extracted.

(**H**) Boxplots depicting log normalized expression of *HOXA9* and *MEIS1*, respectively. The whiskers represent standard deviation, edges depict the inter-quartile ranges, and the black center line the median. A single or double asterisk signifies a p-value < 0.05 or p-value < 0.01, respectively (one-way ANOVA).

(**I**) Principal component analysis plot of iMonocytes and iMLL-AF9 cells ATAC-seq reads .

(**J**) Pie charts visualizing the fraction of ATAC peaks that reside in a particular genomic region.

(**K**) UCSC genome browser illustration of HCB1 MLL-AF9-HA-FKPB12 and MLL-AF9 degraded (3h) ChIP-seq regions of predicted important TFs.

(**L**) Boxplots depicting the average log normalized expression of MLL−AF9−associated core set genes in primary MLL-AF9 AML (TARGET AML cohort). The whiskers represent standard deviation, edges depict the inter-quartile ranges, and the black center line the median. A single or double asterisk signifies a p-value < 0.05 or p-value < 0.01, respectively.

(**M**) Similar to (L), except using the BeatAML cohort.

Supplementary Figure S2. Unsupervised clustering AML cohorts using MLL-AF9 core genes, and single-cell cluster annotation.

(**A**) Radial dendrogram illustrating unsupervised hierarchical clustering using only expression of MLL−AF9−associated core set genes in primary MLL-AF9 AML (TARGET AML cohort).

(**B**) Similar to (A), except using the BeatAML cohort.

(**C**) Expression heatmap depicting a number of established myeloid cell type markers.

(**D**) Donut charts visualizing the fraction of cells per cluster for iMonocyte and iMLL-AF9 conditions.

(**E**) Expression heatmap depicting a number of angio-/vasculogenic markers.
